# BDNF as a Promising Therapeutic Agent in Parkinson’s Disease

**DOI:** 10.3390/ijms21031170

**Published:** 2020-02-10

**Authors:** Ewelina Palasz, Adrianna Wysocka, Anna Gasiorowska, Malgorzata Chalimoniuk, Wiktor Niewiadomski, Grazyna Niewiadomska

**Affiliations:** 1 Mossakowski Medical Research Centre Polish Academy of Sciences, 02-106 Warsaw, Poland; 2 Nencki Institute of Experimental Biology Polish Academy of Sciences, 02-093 Warsaw, Poland; 3 Faculty in Biala Podlaska, Jozef Pilsudski University of Physical Education in Warsaw, 21-500 Warszawa, Poland

**Keywords:** brain-derived neurotrophic factor, neurodegeneration, Parkinson’s disease, physical exercise, neuroprotection, PD therapy

## Abstract

Brain-derived neurotrophic factor (BDNF) promotes neuroprotection and neuroregeneration. In animal models of Parkinson’s disease (PD), BDNF enhances the survival of dopaminergic neurons, improves dopaminergic neurotransmission and motor performance. Pharmacological therapies of PD are symptom-targeting, and their effectiveness decreases with the progression of the disease; therefore, new therapeutical approaches are needed. Since, in both PD patients and animal PD models, decreased level of BDNF was found in the nigrostriatal pathway, it has been hypothesized that BDNF may serve as a therapeutic agent. Direct delivery of exogenous BDNF into the patient’s brain did not relieve the symptoms of disease, nor did attempts to enhance BDNF expression with gene therapy. Physical training was neuroprotective in animal models of PD. This effect is mediated, at least partly, by BDNF. Animal studies revealed that physical activity increases BDNF and tropomyosin receptor kinase B (TrkB) expression, leading to inhibition of neurodegeneration through induction of transcription factors and expression of genes related to neuronal proliferation, survival, and inflammatory response. This review focuses on the evidence that increasing BDNF level due to gene modulation or physical exercise has a neuroprotective effect and could be considered as adjunctive therapy in PD.

## 1. Introduction

Brain-derived neurotrophic factor (BDNF) belongs to neurotrophins (NTs)—a family of proteins that support the function of the central nervous system (CNS). Neurotrophins are synthesized mainly in CNS [1], but also in the non-neuronal peripheral cells such as T and B lymphocytes, monocytes [2], vascular endothelial [3], and smooth [4] and skeletal muscle cells [5]. BDNF expression was confirmed in the hippocampus, frontal cortex, midbrain, amygdala, hypothalamus, striatum (ST), pons, and medulla oblongata [6,7]. BDNF plays a key role in the development of the nervous system by affecting cell differentiation, neuronal development, growth and survival, neurogenesis, synaptogenesis, and synaptic plasticity [7,8,9,10]. Furthermore, it was shown that neurodegenerative and neuropsychiatric diseases may be partially caused by defects in synaptic plasticity associated with the insufficient neuronal supply of BDNF and other neurotrophic factors [11,12,13]. For this reason, there is a need to search for new strategies to increase the BDNF level as a tool in prevention and therapy of neurological diseases.

## 2. Brain-Derived Neurotrophic Factor—Regulation of Synthesis, Activation of Specific Receptors, Location and Function in the Nervous System

The broad spectrum of processes controlled by BDNF can be explained by the specificity of its multistage synthesis, progressing through several intermediate biologically active isoforms that bind to different types of receptors, and can trigger several signaling pathways [14].

The *Bdnf* gene is located on chromosome 11 and contains 9 promoters that can initiate transcription of 24 transcripts, each containing an alternative 5′ noncoding exon spliced to a 3′ coding exon that comprises the entire open reading frame for the BDNF protein [15]. The pre-proBDNF precursor is synthesized in the endoplasmic reticulum and then transported to the Golgi apparatus, where the preregion sequence is cleaved to produce the proBDNF isoform. Then, proBDNF may be converted into mature BDNF (mBDNF) in the trans-Golgi network by the subtilisin-kexin family of endoproteases such as furin or in intracellular vesicles by convertases [14,16]. Extracellular formation of mBDNF is catalyzed by plasmin and matrix metalloproteases. Studies have shown that proBDNF can be also cleaved to release segment longer than mBDNF, which is called truncated BDNF and whose physiological function is largely unknown [15]. The balance between proBDNF and mBDNF depends on the stage of brain development and on the brain region. A higher level of proBDNF is observed during brain development, while mBDNF exerts neuroprotective activity and promotes synaptic plasticity during adulthood [17,18,19,20,21].

Recently, it has been reported that synthesis of BDNF may be affected by 20–22 nucleotide noncoding RNA molecules called microRNAs (miRNAs). MiRNAs bind to 3′ untranslated regions (3′UTR), of target mRNAs and promote their degradation or suppress their translation into proteins, thereby silencing gene expression [22,23,24]. For example, in vitro studies have shown that miRNA-26a and miRNA-26b target 3′UTR of BDNF mRNA in HeLa cells [25], while miRNA-140 and miRNA-211 target the 3′UTR of BDNF mRNA in human astrocyte cultures [26,27]. Furthermore, elevated brain level of miR-206 was reported in the mouse model of Alzheimer’s disease (AD), whereas its reduction enhanced the synthesis of BDNF and improved memory function [28]. A study performed by Li et al. [29] has shown a diminished level of BDNF and an elevated level of miR-132 and miR-182 in the serum of patients with depression compared with healthy controls, which suggest that these miRNAs inhibit BDNF synthesis. Interestingly, Mellios et al. [22] have reported an increased level of several different miRNAs in human prefrontal cortical tissue that corresponded directly with subjects’ age and inversely with a decreased level of BDNF. In turn, a recent study has demonstrated that inhibition of miR-103a can block the activation of astrocytes in the hippocampus and reduce the pathological injury of neurons of epilepsy rats [30]. 

BDNF level in the peripheral tissues, brain, and blood may be also affected by gene polymorphism. BDNF gene polymorphism gives rise to a substitution of methionine for valine at position 66 (Val66Met) in proBDNF, causing a decreased dendritic distribution, reduced BDNF transport to secretory granules, and low activity-dependent secretion of BDNF [31,32]. An ethnicity-specific meta-analysis showed that Val66Met may increase susceptibility to PD in Europeans, but not in Asians, and increase the risk of suicidal behaviors in Asian and Caucasian populations [33].

ProBDNF and mBDNF exert their biological activity by binding to two types of cell surface receptors, the Trk tyrosine kinases and the p75 neurotrophin receptor (p75NTR) [34]. Interestingly, they often have an opposite effect on survival, differentiation, growth, and apoptosis of neurons; thus, the proBDNF/mBDNF balance is an important factor inf the regulation of many processes in CNS [35,36]. mBDNF binds with a high affinity to the TrkB receptor, while proBDNF has a greater affinity to the p75NTR, through its mature domain and to the sortilin receptor through the prodomain. The proBDNF/p75/sortilin complex leads to the activation of c-Jun N-terminal (JNK), Ras homolog gene family member A (RhoA), and nuclear factor kappa B (NF-κB) signaling pathways, which trigger neuronal apoptosis, neuronal growth cone development, and neuronal survival, respectively [14].

Upon mBDNF binding, the TrkB receptor is dimerized and autophosphorylated at tyrosine residues in its intracellular domain. Then, tyrosine residues (Tyr 515 and Tyr 816) located in the juxtamembrane region and in the carboxyl terminus of the receptor [37,38] become phosphorylated, which leads to activation of Ras, phosphatidylinositol 3-kinase (PI3K), phospholipase C-γ (PLC-γ), and their downstream effectors [39]. The downstream effectors include Ras stimulation of mitogen-activated protein kinase (MAPK) pathways, PI3K stimulation of protein kinase B (Akt), and PLC-γ1-dependent generation of inositol trisphosphate (IP3) and diacylglycerol (DAG) that results in mobilization of Ca^2+^ stores and activation of Ca^2+^ and DAG-regulated protein kinases [37,40]. 

**Figure 1:**
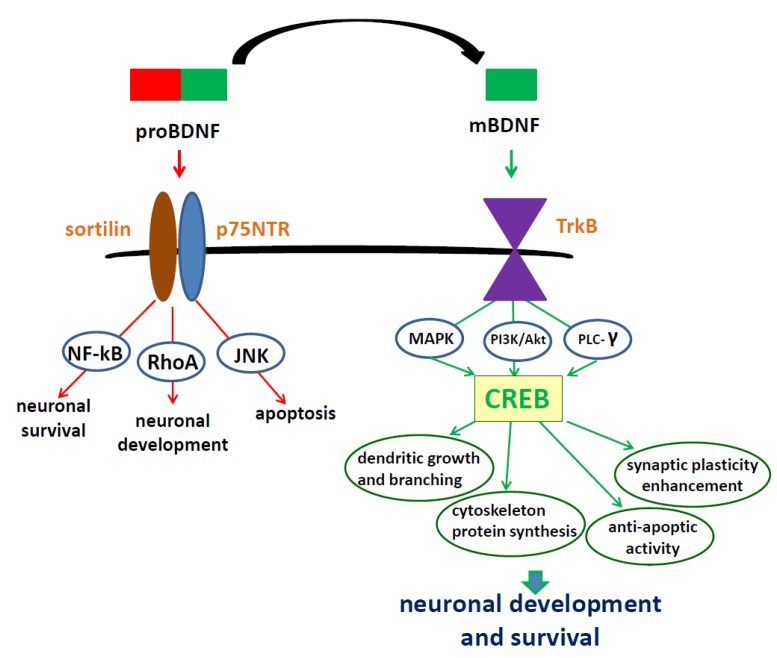
Signaling cascades activated by interaction of BDNF isoforms with two types of cell surface receptors, the p75 neurotrophin receptor and TrkB receptor. proBDNF has a greater affinity for the p75 receptor. The pro-BDNF/p75/sortilin complex leads to activation of JNK, RhoA, and NF-ĸB signaling pathways which promote processes such as apoptosis, neuronal growth cone development, and neuronal survival. The mBDNF/TrkB receptor complex triggers activation of three signaling pathways—MAPK, PI3K/Akt, and PLC-γ—that, in turn, activate the transcription factor CREB and transcription of genes responsible for development and survival of neurons. proBDNF— precursor of brain-derived neurotrophic factor, mBDNF—mature brain-derived neurotrophic factor, TrkB—tropomyosin receptor kinase B, JNK—c-Jun N-terminal kinases, RhoA—Ras homolog gene family member, NF-ĸB—nuclear factor kappa B, MAPK—mitogen-activated protein kinase, PI3K—phosphatidyl inositol-3 kinase, PLC-γ—phospholipase C-γ, CREB—cAMP response element-binding protein.

BDNF-TrkB signaling pathways regulate multiple events, such as dendritic growth, pine maturation and stabilization [41,42,43], development of synapses [44,45], learning- and memory-processes-dependent synaptic plasticity [40,46,47], apoptosis, and survival of neurons [48,49,50,51]. The intracellular signaling cascades and physiological function of BDNF are key to understanding the mechanisms underlying the neuroprotective properties of this neurotrophin, enabling proper cell functioning and survival (Figure 1).

## 3. Role of BDNF in Neurodegeneration and Neuroregeneration

Neurotrophic factors have been extensively investigated in the context of neurodegenerative diseases. The alterations in the regulation of specific neurotrophic factors and their receptors seem to be involved in neurodegeneration. Neurotrophins prevent cell death and support neuronal proliferation and maturation, enhancing the growth and function of affected neurons in AD and PD [52,53]. In current therapies for AD and PD which focus on prevention of neurodegeneration, application of neurotrophic factors has emerged as one of the therapeutic approaches in early, middle and even late stages of these disorders. Even though studies in animal models are promising, their effectiveness in clinical studies remains unclear [53,54].

By activating the IP3K/Akt kinase pathway, neurotrophins inhibit processes that elicit cell death [55,56,57]. It was found that the decrease in expression of NTs, especially BDNF observed in the aging process and in neurodegenerative diseases, may contribute to degeneration and death of neurons [58]. A decrease in BDNF levels in the blood and brain was observed in patients with depression or suffering from PD and AD [59,60,61,62]. Decreased BDNF concentration in serum and brain is accompanied by an increase in degeneration of dopaminergic neurons in PD, which leads to movement disorders, cognitive deficit, and mental disorders [63,64,65,66] and also correlates with memory impairment in AD [67,68]. There are data indicating that a decrease in BDNF levels in PD may contribute to overexpression of alpha-synuclein (ASN) and inhibition of dopamine (DA) synthesis [69,70,71]. Moreover, it was reported that ASN overexpression downregulated BDNF transcription and impaired BDNF trafficking in neurons [53]. BDNF participates in the regulation of tyrosine hydroxylase (TH), which is also reduced in PD, consequently leading to motor disorders [72,73]. In addition, Ziebell et al. [74] have found that in patients with striatal dopaminergic neurodegeneration, serum BDNF levels decrease along with loss of the striatal dopamine transporter (DAT). Silencing the gene encoding BDNF in mice resulted in the loss of dopaminergic neurons, which confirms the role of BDNF in protecting neurons against injury and degeneration [72,75,76]. There are studies showing that glial cell-derived neurotrophic factor (GDNF) can also prevent the degeneration of dopaminergic neurons in PD [77,78].

The neuroprotective effect of BDNF is the result of activation of the TrkB/MAPK/ERK1/2/IP3K/Akt pathway, which leads to attenuation of apoptosis, glutamate, and nitric oxide (NO) neurotoxicity and cell damage caused by oxidative stress [79]. An increase in oxidative stress, glutamate neurotoxicity, NO production, and the process of apoptosis are observed in PD [80] and other neurological disorders [81,82].

A key role of BDNF in the deterioration of motor and cognitive abilities in PD may arise from experiments on animal models, where BDNF level was decreased due to genetic modification. The knockout of the *Bdnf* in midbrain–hindbrain in WNT-BDNF^KO^ mice resulted in motor impairments (hind limb clutching and poor rotarod performance) [72]. In turn, 6-8-week-old *Bdnf*+/− mice showed worse performance in Morris Water Maze than control animals, whereas 10-month-old *Bdnf*+/− animals did not learn at all, suggesting that the BDNF may alter learning abilities [83].

## 4. BDNF as a Promising Compound in the Therapy of Parkinson’s Disease

The body of data suggesting a relation between the decline of BDNF level and progression of PD is growing steadily [62,84,85,86,87]. The fact that there is currently no cure for PD highlights the need to seek new therapeutic agents that will make the treatment of PD more effective. Given the properties of BDNF, its application for treatment of neurodegenerative diseases, including PD, seems promising.

### 4.1. Upregulation of BDNF Signaling through Direct Injection and Gene Therapy

#### 4.1.1. Study in PD Animal Models

In the animal models of PD, BDNF brain content was augmented through direct injection of BDNF [88,89], gene transduction by viral vectors [90,91,92,93,94], or delivery via nonviral carriers [95,96] and also via secretion from genetically engineered cells [97,98,99,100,101]. The effects of BDNF treatment were studied in two research schemes in which BDNF was administered before and after the induction of PD.

In the vast majority of studies with BDNF administration before induction of parkinsonism, the research results revealed at least partial prevention of neuronal cell loss [89,90,92,93,99,101]. BDNF increased survival of Nissl- or neuronal-nuclei-stained neuronal cells [92,99,101] and dopaminergic neurons [89,92,93,99] in substantia nigra (SN) and protected dopaminergic projections to ST [92,93]. Moreover, BDNF administration increased DA level in SN [92,100] and ST [88,92] and restored DA uptake in ST [101]. However, the reports are not consistent; some have shown no increase in neuronal survival when BDNF was applied before intoxication with 6-hydroxydopamine (6-OHDA) in PD animal model [90,91].

Similarly controversial results were obtained when induction of parkinsonism by 6-OHDA lesion was followed by BDNF treatment. There seemed to be no recovery of the dopaminergic neurons number in SN [92,95,97,98]. However, Kim et al. [92] demonstrated dopaminergic axon regrowth when the *Bdnf* gene was transduced 6 weeks after the axonal lesion. Moreover, BDNF treatment of brain slices from 1-methyl-4-phenyl-1,2,3,6-tetrahydropyridine (MPTP)-treated mice was able to restore the impaired synaptic plasticity [102]. In addition, Hernandez-Chan et al. [95] observed an increase in the striatal level of DA, while in SN there was no recovery of DA content. Therefore, it seems that even though BDNF does not induce neurogenesis, it exerts a positive influence on the remaining neurons. In contrast, Razgado-Hernandez et al. [96] noticed that the combined administration of agonist of D3 subtype of the dopamine receptor and gene-delivered BDNF are able to markedly rescue dopaminergic neurons in SN and dopaminergic projections to ST. Additionally, the treatment fully recovered the number of dopaminergic spines [96]. Furthermore, virus-derived BDNF upregulated dopamine D1 receptor-dependent cyclicAMP/protein kinase A (cAMP/PKA) signaling cascade [94], which was shown to alter impaired fear extinction in PD mice [103]. Interestingly, BDNF modulated also serotonergic system activity, which, together with dopaminergic and noradrenergic system, is involved in motor control. BDNF elevated serotonin (5-hydroxytryptamine, 5-HT) terminal sprouting in ST and globus pallidus measured as an increase in serotonin fibers density [94].

In the case of alternations in behavior, the studies on monkeys revealed significantly less severe symptoms of PD in animals treated with BDNF [89]. Moreover, Kim et al. [92] observed a decreased amphetamine-induced ipsiversive bias in rotational behavior associated with the protection of dopaminergic neurons [92]. Despite the lack of dopaminergic neuroprotection, Klein et al. [90] and Yoshimoto et al. [98] noticed a significant decline in amphetamine-induced ipsiversive rotational bias indicative of the restoration of DA neurotransmission on the injected side. In turn, Sun et al. [91] reported that combined treatment with BDNF and GDNF resulted in a greater reduction in the ipsiversive bias as compared to BDNF treatment.

It was also shown that the BDNF administration recovers spontaneous motor behavior in the rat model of PD [95]. Combined treatment with a D3 receptor agonist and BDNF in 6-OHDA lesioned rats led to the stabilization of gait parameters, accelerated recovery of motor coordination and balance, and full recovery from muscle rigidity [96]. Contrastingly, Lucidy-Philipi et al. [97] reported no recovery of the rotational behavior in BDNF-treated parkinsonian animals.

**Figure 2:**
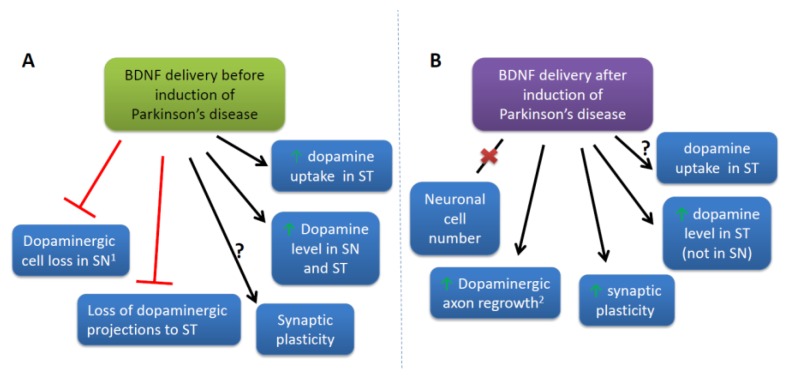
Summary of the major effects of BDNF delivery through direct injection and gene therapy before and after the induction of Parkinson’s disease (PD) in animal models. (**A**) BDNF signaling upregulation before the induction of PD prevented dopaminergic cell loss in SN and the loss of dopaminergic projections to ST. BDNF stimulation elevated the DA level in SN and ST, and DA uptake in ST. To the best of our knowledge, studies concentrating directly on synaptic plasticity were not conducted. (**B**) BDNF delivery after induction of PD did not alter the number of dopaminergic neurons; however, it induced dopaminergic axon regrowth, increased synaptic plasticity, and elevated the DA level in ST, but not in SN. To our knowledge, DA uptake was not studied in this BDNF administration paradigm. Exceptions to the rules: ^1^ Lack of neuronal cells preservation by BDNF treatment before PD induction [90,91]. ^2^ Lack of effect on TH+ fibers in ST by BDNF treatment after PD induction [97,98]. BDNF—brain-derived neurotrophic factor, DA—dopamine, PD—Parkinson’s disease, SN—substantia nigra, ST—striatum.

In summary, BDNF treatment in animal models seemed to enhance dopaminergic neurons survival when administered before induction of PD or at least caused partial recovery of dopaminergic transmission despite lack of neurogenesis (Figure 2).

#### 4.1.2. Study in Humans 

Neurotrophins are challenging candidates for delivery into CNS due to the short in vivo half-life, poor bioavailability, and marginal permeability through the blood-brain-barrier (BBB). Furthermore, an important issue regarding chronic neurodegenerative disorders is the duration of treatment, which may last for years [104].

The first clinical trials that investigated the effect of BDNF administration in neurodegenerative diseases were performed in patients with amyotrophic lateral sclerosis (ALS) [105,106]. A study with subcutaneous administration of recombinant methionyl human BDNF (r-metHuBDNF) did not demonstrate a statistically significant effect of BDNF on the survival of these patients and did not replicate the beneficial effect of BDNF obtained in I and II phase of the study [105]. The purpose of the subsequent study was to determine the conditions for direct delivery of r-metHuBDNF to the cerebrospinal fluid (CSF) by a lumbar intrathecal catheter. Twenty-five patients with probable or definite ALS were treated with either r-metHuBDNF (25, 60, 150, 400, or 1000 mg/day) or placebo in a 12-week, randomized, double-blinded, sequential, dose-escalation study. This clinical trial showed that BDNF treatment with doses of 150 mg/day was well tolerated and provided BDNF concentration in CSF that could be neuroprotective, but the number of patients included in the study was too small to make a conclusion about the efficacy of the treatment [106]. Clinical trials using NTs in the treatment of PD are rather limited to GDNF administration instead of BDNF. Some of them report the direct, beneficial, but transient effect of GDNF on DA function [107,108], and others show no effect of GDNF treatment on PD progression [109,110] and many side effects after its administration [110].

The fact that clinical trials have met with little success may be explained taking into account that successful delivery of therapeutic molecules should evade (i) rapid protein elimination from the cerebral circulation owing to enzymatic degradation, (ii) capture by the reticuloendothelial system, (iii) macromolecular accumulation in nontargeted tissues, and (iv) undesired immune responses [111]. Furthermore, currently, it is not possible to ensure that BDNF acts on precise target regions without affecting the function of neighboring areas [112].

Gene therapy is a strategy for enhancing BDNF expression in CNS. The choice of vector determines the technique used for its delivery. When a vector is peripherally administered, it must be able to cross the BBB with acceptable tissue specificity [113]. The most often used viral vectors infect the host cells, deliver a new gene, and lead to biosynthesis of a particular functional protein [114,115]. Theoretically, in vivo gene delivery can meet the requirements for safe and effective growth factor delivery to the brain and circumvent delivery to the brain with minimal (or no) spreading to nontargeted regions and reduced likelihood of protein instability [104]. Animal research on gene therapy additionally demonstrates that viral vectors successfully transduced neurons and provided the durable expression of BDNF [116,117,118,119]. 

To date, no clinical research on BDNF gene therapy has been performed. Thus, it is unknown whether the efficacy of neurotrophic factors gene therapy can be achieved. Current gene therapy trials in PD are focused on adenosine A_2A_ antagonists [120], glutamic acid decarboxylase [120], GDNF [120,121], aromatic L-amino acid decarboxylase [120,122], and neurturin [123,124]. Out of these several approaches, only adenosine A2A antagonists show promising results in the management of motor complications in advanced stages of PD. A credible explanation for the low effectiveness of this approach may lie in the fact that dopaminergic neurons are dying or have already died [113].

### 4.2. Stimulation of Endogenous BDNF Level by Physical Effort

#### 4.2.1. Study in PD Animal Models

BDNF production was shown to be stimulated by both voluntary [75] and forced physical activity [125,126,127,128,129,130,131,132,133]. BDNF upregulation was observed in midbrain [125], SN [126,128,129], ST [127,129,131,133], hippocampus [133], and cortex [132,133]. Tuon et al. [131] reported an attenuated decline of proBDNF content in 6-OHDA-induced PD rats trained for 60 days before lesion. In our study, we also noted a higher BDNF and GDNF immunostaining intensity in nigrostriatal areas of MPTP-treated mice which started treadmill training before (preceding training) and after (follow-up training) intoxication, as compared to control groups both sedentary and exercised [125]. Similarly, enzyme-linked immunosorbent assay showed a significant increase in BDNF in both MPTP preceding training and follow-up training groups as compared to both controls and MPTP sedentary group in the midbrain including SN. In ST, training increased BDNF concentration in both MPTP groups of trained animals and the control trained group as compared to sedentary control and MPTP animals.

BDNF brain level increase due to exercise was accompanied by several other effects. In animal models of PD, exercise was able to increase the number of TH-ir neurons in SN [125,126,127,128,129,130,134], the TH protein level in SN [126,128] and ST [128,134], and TH-ir fiber intensity in ST [127,128]. Physical activity also led to a normalization of the DA content in SN and/or ST [75,126,129,130,132,133] and of 3,4-dihydroxyphenylacetic acid (DOPAC) level in ST [129,133], to a slight increase in the homovanillic acid (HVA) content in ST [133] and an elevation in the DAT level in ST [126,133]. HVA and DOPAC are DA metabolites, whereas DAT is responsible for DA reuptake from synapses. All these substances are the markers of the dopaminergic system and drop in the course of PD [135,136,137]. Furthermore, our study revealed a recovery of the intensity of vesicular monoamine transporter 2 (VMAT2) immunostaining in SN in exercised MPTP mice, indicative of the increase in the number of dopaminergic neurons [125].

In another study, it was shown that exercise increased TrkB content in SN [129]; however, in yet another study, TrkB content was significantly decreased in ST even below the level in the sedentary PD mice [131]. The authors suggest that the decreased TrkB level may be related to changes in synaptic plasticity [131]. In the case of the hippocampus, Tuon et al. [131] noticed a significant restoration of the relative TrkB content in 6-OHDA mice after 60 days of treadmill running. An injection of K252a, an inhibitor of the BDNF receptor, was able to obliterate the protective effect of exercise on dopaminergic neuron number in SN [128,129] and prevent the rise of TH protein level in SN and ST [128]. It seemed that the BDNF-TrkB pathway upregulation by exercise shielded the dopaminergic neurons in SN from lipopolysaccharide-induced degeneration [129] and 6-OHDA lesion in the animal model of PD [128].

Moreover, in MPTP mice with 18 weeks of pretraining, Lau et al. [126] showed protection of mitochondrial integrity and respiratory function in ST as compared to sedentary MPTP mice. In addition, da Costa group [133] also observed the drop in oxidative stress, which was measured as nitrite content and lipid peroxidation level.

The active glycogen synthase kinase 3 beta (GSK-3β) was shown to mediate the MPTP- and 6-OHDA-induced neuronal cell death [138,139,140,141]. Choe et al. [134] observed deactivation of glycogen synthase kinase 3 beta (GSK-3β) by its phosphorylation in both the lesioned and control side in 6-OHDA-induced rats, which trained for 16 days on the treadmill before lesion. It is possible that the GSK-3β deactivation may be caused by BDNF. Binding of BDNF to TrkB receptor leads to deactivation of GSK-3β by its phosphorylation, which in turn promotes cell survival [142].

Gerecke et al. [143] reported that heterozygous deletion of the BDNF gene (*Bdnf*+/− mice) led to a lack of exercise-induced dopaminergic neuroprotection. Moreover, trained *Bdnf*+/− animals showed changes in protein expression related to energy metabolism, cytoskeleton, glycolysis, amino acid transport, synthesis and metabolism, and smaller changes in cytoplasmic signaling molecules and regulatory factors. Therefore, it seems that biallelic expression of BDNF is indispensable for the protective effect of exercise.

In addition, it was shown that, in animal models of PD, training is able to improve motor performance, namely movement and balance on a balance beam [126] and motor memory in rotarod test [129,133]. Moreover, physical exercise increased spontaneous locomotor activity and subthreshold L-DOPA-induced activity [133] and decreased depressive-like behavior, measured as immobility, in open field test [131]. Furthermore, physical training decreased contralateral bias in cylinder test [127] and ipsilateral rotations in amphetamine-induced rotational test [127,134] and reduced rotational asymmetry in apomorphine-induced rotational test [128,131,133]. Additionally, the use of BDNF receptor inhibitor K252a reversed the protective effect of exercise on rotational bias [128].

**Figure 3:**
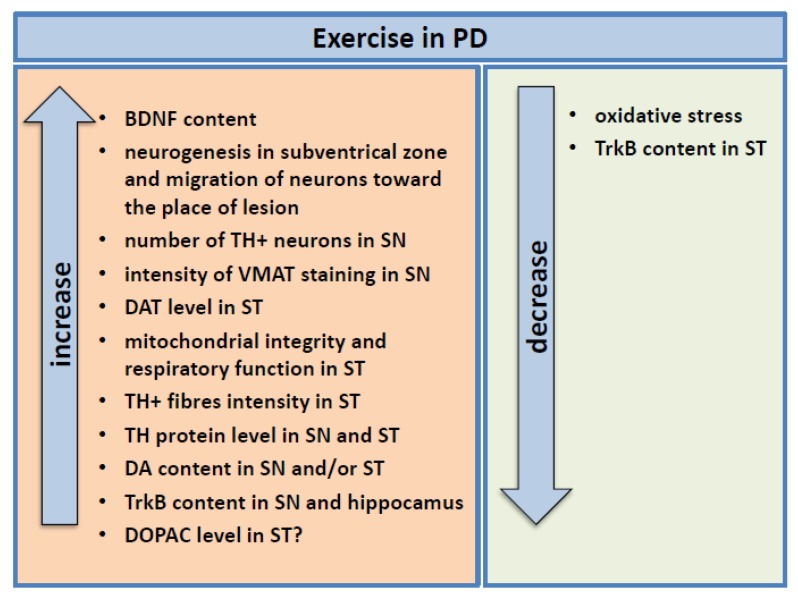
The molecular and physiological changes caused by exercise in animal models of Parkison’s disease. Physical effort led to an increase in BDNF level, TH-ir cell number and TH protein content in SN, TH-ir fibers intensity in ST, and DA content in SN and/or ST. The results from DOPAC content analysis in the brain were contradictory. Training also increased the level of TrkB in SN and hippocampus, but not in ST. In addition, exercise increased the DAT level in ST, VMAT staining intensity in SN. Physical activity was able to maintain mitochondrial integrity and respiratory function in ST, elevate neurogenesis in subventrical zone and migration of neurons toward the place of lesion, and decrease oxidative stress. BDNF—brain-derived neurotrophic factor, TrkB—tropomyosin receptor kinase B, SN—substantia nigra, ST—striatum, DA—dopamine, TH—tyrosine hydroxylase, DOPAC—3,4-Dihydroxyphenylacetic acid, DAT—dopamine active transporter, VMAT—vesicular monoamine transporter.

The aforementioned results from animal models of PD may indicate the key role of BDNF in exercise-driven neuroprotection (Figure 3). It seems that blocking BDNF signaling by inhibition of the TrkB receptor revokes the beneficial effects of physical exertion.

#### 4.2.2. Study in Humans

The results of numerous studies show that regular long-term physical activity prevents the occurrence of neurodegenerative diseases in the elderly. Exercise improves mobility and increases muscle strength [144]. In addition, exercise therapy in the form of endurance training, resistance training, intensive mixed training, or high-intensity interval training (HIIT) resulted in a reduction of clinical symptoms in PD [145,146,147,148,149,150,151,152]. Long-term resistance and HIIT training reduced muscle tone and stiffness and had a positive effect on body balance in people with PD, osteoarthritis, and AD [153,154,155,156]. In addition, these forms of activity led to a reduction of movement disorders and a decrease in cognitive deficit in patients with PD [157]. Studies in healthy subjects have provided evidence that single aerobic exercise causes an increase in BDNF levels in plasma and serum [158,159]. It was also found that a few weeks or several months of physical training causes an increase in the initial BDNF level compared to the state before training, both in healthy subjects and PD patients [150,160,161,162]. This increase depends on the intensity and duration of the training process [163].

A systematic and critical literature search conducted by Knaepen et al. [160] demonstrated that acute aerobic, but not strength, exercise increased basal peripheral BDNF concentrations in healthy subjects, although the effect was transient. Also, a study performed by Schmolesky et al. [164] has indicated a significant increase in serum BDNF in adult human males after aerobic exercise, whereof a 40 min-long vigorous exercise (80% heart rate reserve) was most likely to produce a significant BDNF elevation. In most studies using acute protocols of training, BDNF concentration returned to baseline within 10–60 min postexercise, probably in response to greater tissue absorption [160]. The number of studies regarding the BDNF level in CSF in PD is limited, and their results are contradictory [165,166]. Interestingly, a meta-analysis performed by Hirsch et al. [167], in which results regarding 100 ambulatory patients with idiopathic PD (Hoehn/Yahr ≤3) were reviewed, found improvements in BDNF blood concentration levels after physical activity in all six analyzed studies.

## 5. Possible Mechanisms Underlying the Protective Effect of BNDF Induced by Exercise

The results obtained so far indicate that the increase in peripheral BDNF after aerobic or strength exercise is a temporary effect. It was also shown that a dose-response relationship exists between the intensity of the exercise and peripheral BDNF concentration [145]. The source of circulating BDNF in response to exercise is uncertain and widely discussed in the literature. It has been speculated that the exercise-induced increase in blood BDNF level originates partially from the contracting muscle cells. Although neurons in muscles produce BDNF as a result of exercise, it does not enter the bloodstream. In their studies, Matthews et al. [168] and Pratesi et al. [169] found an increase in BDNF mRNA expression in skeletal muscle in humans after physical effort and after electrical stimulation, but no release into the blood was observed. This concept is supported by data acquired both in vivo and with the use of a muscle cell culture model [168,169,170]. An increase in BDNF serum levels after exercise may be explained by the assumption that the brain is the main source of exercise-induced BDNF circulating in the blood [171,172,173]. Although the permeability of BBB for BDNF is very limited, there is evidence for a release of BDNF from the brain during exercise [174,175], which may reflect an association between peripheral and central BDNF levels and highlight plausibility of the hypothesis according to which the source of increased BDNF concentration due to exercise would be CNS. This concept is supported also by the fact that platelets do not produce BDNF. Platelets store circulating BDNF secreted by the brain, which they then can release back into the bloodstream [173].

In a period of about minutes to one hour after cessation of exercise, the level of peripheral BDNF concentration returns to the baseline [174,176,177,178]. Interestingly, after about 2–3 h since the end of the exercise, a significant drop below the baseline level of this neurotrophin has been observed [178]. The explanation of this phenomenon was attempted by Knaepen et al. [160]. They suggested that physical effort leads to a transient increase in BDNF synthesis in cells, from where its excess can be released into the bloodstream, resulting in an increase in peripheral BDNF levels. Then, BDNF can be absorbed by central and/or peripheral nerve tissues, in which it can induce neurotrophic and neuroprotective processes. This cycle, at least in part, explains the rapid return of BDNF concentration during postexercise restitution to values observed before exercise. Nevertheless, it seems that increase in BDNF is linearly associated with exercise intensity. Regular and systematic physical effort of moderate intensity may constantly raise BDNF concentration in the brain. Even though a single exercise causes a BDNF increase in the blood of patients with PD, it is short-lived and is not associated with an improvement in the neurological status of the patients. Studies using an animal model of parkinsonism have provided evidence that only the repetition of medium- or high-intensity physical exercise for several weeks or a continuous training process causes an increase in BDNF [179] and TrkB receptor levels [131,180] in the brain regions responsible for motor activity in rats.

Under the influence of the training process, increased BDNF expression in the hippocampus, cerebral cortex, ST, brainstem, and spinal cord was observed [131,174,179,181,182]. Animal studies have shown that long-term endurance training increased both TrkB receptor and TH expression in structures involved in extrapyramidal movement regulation, i.e., in the ST and midbrain [128,129,179,183,184,185]. As previously mentioned, the key element of neurotrophin-related neuroprotection is the IP3/Akt kinase pathway [186]. Data were also presented which indicate that physical activity may be neuroprotective by activating the PGC1a/FNDC5/BDNF/ERK1/2 pathway [187,188]. In addition, exercises increase transcription of peroxisome proliferator-activated receptor gamma coactivator 1-alpha (PGC1α), a protein that regulates mitochondrial biogenesis, which, in complex with estrogen-related receptor alpha, can stimulate the expression of myokine, the fibronectin type III domain-containing protein 5 (FNDC5) in the brain [187]. After cleavage of the signal peptide and cleavage of the site flanking the fibronectin domain, irisin glycopeptide is produced that triggers BDNF expression [189,190]. Thus, the increase of PGC1a expression may in turn increase BDNF level.

**Figure 4:**
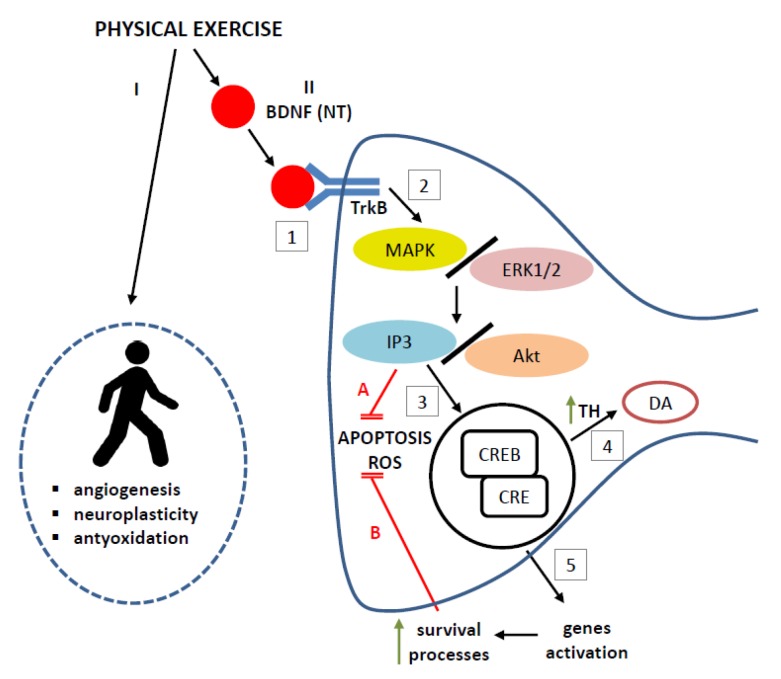
Systemic (I) and central nervous system (II) responses to physical exercise. (I) Physical exercise promotes angiogenesis and neuroplasticity, and anti-oxidation counteracts oxidative stress. (II) Physical exercise increases BDNF affinity to the TrkB receptor (1) enhancing a cascade of intracellular signals, including MAPK/ERK1/2–IP3/Akt pathway (2) that inhibits apoptosis and free radical release—ROS (A) on the one hand, and phosphorylation of transcription factor CREB on the other (3); the latter, by attaching to the CRE elements in the cell nucleus, increases the transcription of the tyrosine hydroxylase gene (4) responsible for conversion of tyrosine to L-DOPA, from which DA is formed, and transcription of genes (5) that promote the survival processes, thereby blocking apoptosis and inhibiting the formation of ROS (B). Akt—Akt enzyme, also known as protein kinase B, BDNF—brain-derived neutrophic factor, CREB—cyclicAMP-response element-binding protein, DA—dopamine, ERK—extracellular signal-regulated kinases, IP3—inositol trisphosphate, L-DOPA—levodopa, MAPK—mitogen-activated protein kinase, ROS—reactive oxygen species, TrkB—tropomyosin receptor kinase B.

BDNF binds to the TrkB receptor on the surface of the neurovascular unit and triggers a cascade of intracellular signals, transmitted primarily by MAPK/ERK1/2 and calcium and calmodulin-dependent protein kinase II (CaMKII), which are responsible for phosphorylation of cAMP-response element-binding protein (CREB, transcription factor, key for long-term neuronal plasticity). CREB binds to the appropriate sequence in the TH gene promoter, thereby increasing TH transcription [191,192]. Among the mechanisms related to exercise-dependent neuroplasticity, CREB is directly involved in regulating TH expression in PD [193,194]. Increased TH transcription plays a crucial role in exercise-dependent neuroplasticity. Application of physical exercises causes activation of CREB by different signal transducers, such as MAPKs, CaMK, and N-methyl-D-aspartate receptor (NMDA-R) in both the hippocampal and striatal plasticity [195,196]. An increase in TH enzyme activity, which is involved in the conversion of L-tyrosine to L-DOPA, from which DA is formed, favors the survival of dopaminergic neurons (Figure 4).

Some authors suggest that physical effort mediates BDNF expression through monoaminergic activation, which entails increased adrenergic (noradrenaline) or serotoninergic (5-HT) neurotransmission. Indeed, prolonged physical activity causes an increase in NA and 5-HT activation associated with regulation of synapse plasticity and the signs of neuroprotection in the brain [197,198]. It has also been suggested that NA activation of β-adrenergic receptors may be necessary for exercise-induced BDNF regulation. The G-protein-coupled receptor and MAPK- and IP3K-mediated signaling pathways are indicated as those regulating the NA-dependent expression of the gene encoding BDNF [199,200].

There are also data that show that BDNF participates in the regulation of neuron metabolism, development, and proper functioning in cooperation with insulin-like growth factor-1 (IGF-1) [201]. Some authors indicate that the presence of both IGF-1 and BDNF is required for the viability and proper functioning of neuronal cells [186]. Physical exercise is known to increase the secretion of IGF-I and vascular endothelial growth factor (VEGF) which, together with BDNF, can improve memory by modulating synaptic plasticity, synapses, and neurotransmission in mature neurons [202].

Physical training protects DA neurons in the SNpc against inflammatory insult [179]. The beneficial effects of exercise are due to the activation of BDNF signaling pathway. Chronic exercise may reduce microglial reactivity and inflammation through regulation of multiple metabolic and transcriptional processes [203]. GSK-3 is a major regulator of the balance between the pro- and anti-inflammatory mediators in immune cells, including microglia [204]. GSK-3 stimulates the release of interleukin 1 beta (IL-1β), interleukin 6 (IL-6), and tumor necrosis factor alpha in activated microglia and inhibits the release of anti-inflammatory cytokines like interleukin 10 (IL-10) [205]. Exercise may activate some extracellular signals that are known to inhibit GSK-3, including BDNF [180,206]. It is also possible that, as a result of a prolonged exercise, which increases the synthesis of trophic factors, there is no proinflammatory proliferation and activation of glial cells. This is because dopaminergic neurons protected by NTs do not degenerate and thus do not send signals mobilizing the proinflammatory response.

## 6. Conclusions

BDNF exerts well-documented neuroprotective and neurorestorative effects on dopaminergic neurons, which makes it a promising agent in PD therapy. However, neither direct delivery of exogenous BDNF into the patient’s brain nor attempts to enhance BDNF expression with gene therapy turned out successful. Properly selected physical training may permanently increase BDNF level in blood and brain and, as evidenced in animal models, is to some extent able to protect neurons against neurotoxic assaults. Understanding the way training induces enhancement of BDNF expression and the mechanisms by which BDNF induces neuroprotection and neurorestoration may help design pharmacological treatment of PD and pave the way for novel PD therapies.

## Abbreviations

5-HT	5-hydroxytryptamine, serotonine
6-OHDA	6-hydroxydopamine
AAV2	adeno-associated virus serotype 2 vector
AD	Alzheimer’s disease
Akt	Akt enzyme, also known as protein kinase B
ALS	amyotrophic lateral sclerosis
ASN	alpha-synuclein
BBB	blood-brain-barrier
BDNF	brain-derived neurotrophic factor
CaMKII	calmodulin-dependent protein kinase II
cAMP	cyclicAMP
CNS	central nervous system
CREB	cAMP-response element-binding protein
CSF	cerebrospinal fluid
DA	dopamine
DAG	diacylglycerol
DAT	dopamine transporter
DOPAC	3,4-dihydroxyphenylacetic acid
ERK	extracellular signal-regulated kinases
GDNF	glial cell-derived neurotrophic factor
GSK-3β	glycogen synthase kinase 3 beta
HVA	homovanillic acid
IGF-1	insulin-like growth factor-1
IL-1β	interleukin 1 beta
IL-6	interleukin 6
IP3	inositol trisphosphate
JNK	c-Jun N-terminal kinases
L-DOPA	levodopa
LTP	long-term potentiation
MAPK	mitogen-activated protein kinase
mBDNF	mature BDNF
miRNAs	microRNAs
MPTP	1-methyl-4-phenyl-1,2,3,6-tetrahydropyridine
NF-κB	nuclear factor kappa B
NGF	nerve growth factor
NMDA-R	N-methyl-D-aspartate receptor
NO	nitric oxide
NRTN	neurturin
NT-3	neurotrophin-3
NT-4	neurotrophin-4
p75NTR	p75 neurotrophin receptor
PD	Parkinson’s disease
PET	positron emission tomography
PGC1α	peroxisome proliferator-activated receptor gamma coactivator 1-alpha
PI3K	phosphatidylinositol-3 kinase
PKA	protein kinase A
PLCγ	phospholipase Cγ
RhoA	Ras homolog family member A
ROS	reactive oxygen species
r-metHuBDNF	recombinant methionyl human BDNF
SN	substantia nigra
ST	striatum
TH	tyrosine hydroxylase
TrkB	tropomyosin receptor kinase B
VEGF	vascular endothelial growth factor
VMAT2	vesicular monoamine transporter 2
